# Regulation of shear-induced nuclear translocation of the Nrf2 transcription factor in endothelial cells

**DOI:** 10.1186/1423-0127-16-12

**Published:** 2009-01-22

**Authors:** Chung-Yu Hsieh, Huai-Yu Hsiao, Wan-Yi Wu, Ching-Ann Liu, Yu-Chih Tsai, Yuen-Jen Chao, Danny L Wang, Hsyue-Jen Hsieh

**Affiliations:** 1 Department of Chemical Engineering, National Taiwan University, Taipei 106, Taiwan; 2 Institute of Biomedical Sciences, Academia Sinica, Taipei 115, Taiwan

## Abstract

**Background:**

Vascular endothelial cells (ECs) constantly experience fluid shear stresses generated by blood flow. Laminar flow is known to produce atheroprotective effects on ECs. Nrf2 is a transcription factor that is essential for the antioxidant response element (ARE)-mediated induction of genes such as heme-oxygenase 1 (HO-1). We previously showed that fluid shear stress increases intracellular reactive oxygen species (ROS) in ECs. Moreover, oxidants are known to stimulate Nrf2. We thus examined the regulation of Nrf2 in cultured human ECs by shear stress.

**Results:**

Exposure of human umbilical vein endothelial cells (HUVECs) to laminar shear stress (12 dyne/cm^2^) induced Nrf2 nuclear translocation, which was inhibited by a phosphatidylinositol 3-kinase (PI3K) inhibitor, a protein kinase C (PKC) inhibitor, and an antioxidant agent N-acetyl cysteine (NAC), but not by other protein kinase inhibitors. Therefore, PI3K, PKC, and ROS are involved in the signaling pathway that leads to the shear-induced nuclear translocation of Nrf2. We also found that shear stress increased the ARE-binding activity of Nrf2 and the downstream expression of HO-1.

**Conclusion:**

Our data suggest that the atheroprotective effect of laminar flow is partially attributed to Nrf2 activation which results in ARE-mediated gene transcriptions, such as HO-1 expression, that are beneficial to the cardiovascular system.

## Background

Vascular endothelial cells (ECs) are in direct contact with blood flow and are constantly exposed to blood flow-generated shear stresses. Accumulated data in the literature reveal that laminar shear stress is beneficial for the endothelium [1]. Numerous studies and accumulating microarray data [2-5] indicate that physiological shear stresses produce antioxidant [6], antiapoptotic [7], anti-inflammatory [8], and antiproliferative effects [9,10]. Investigations from our laboratories and others have shown that shear stress inhibits serum-, cytokine-, and hydrogen peroxide-induced responses [11-14]. Shear stresses also initiate cascades of events that are essential for endothelial function. For example, shear stress can stimulate phosphatidylinositol 3-kinase (PI3K) activity [15] which is required for Akt phosphorylation; this helps prevent endothelial apoptosis [7] and contributes to endothelial nitric oxide synthase (eNOS) activation and subsequent nitric oxide (NO) production [16,17]. NO acts as a vasodilator and exerts atheroprotective effects on the endothelium by inhibiting many atherosclerosis-prone events [18-21]. Moreover, a number of antioxidant genes, such as heme-oxygenase 1 (HO-1), NAD(P)H:quinine oxidoreductase-1, and glutathione S-transferase, are upregulated in ECs under laminar shear stress, and the antioxidant response element (ARE) which resides in the promoter regions of these antioxidant genes plays a vital role in their induction [6]. Upregulation of the antioxidant, superoxide dismutase (SOD), by shear stresses can suppress the apoptotic effects induced by other agents [22]. However, the detailed mechanisms of how the antioxidant ability of shear stresses is regulated remain to be elucidated.

Nuclear factor erythroid 2-related factor 2 (Nrf2) is a cap'n'collar (CNC) basic leucine zipper transcription factor [23]. Evidence compiled from in vitro and in vivo studies has shown that Nrf2 is essential for ARE-mediated induction of genes including phase II detoxifying enzymes and antioxidant enzymes [24-26]. Results obtained from experiments exploiting Nrf2-null mice suggest that Nrf2 plays a protective role against xenobiotics, oxidative stress, and cardiovascular injuries [26-28]. A recent study revealed that Nrf2 activation inhibits inflammatory gene expression [29]. The N-terminal domain of Nrf2 is bound to the cytoskeletal-associated protein, Keap1, that negatively regulates Nrf2 by both repressing Nrf2 transcriptional activity [30] and enhancing its rate of proteasomal degradation [31,32] in the cytoplasm. Upon stimulation, Nrf2 dissociates from Keap1 and is translocated into the nucleus to initiate the following transcriptional events [30]. Oxidants and electrophiles are known to stimulate Nrf2 [33,34], and herein we show that hydrogen peroxide (H_2_O_2_), a major reactive oxygen species (ROS), is another Nrf2 stimulator. A recent study demonstrated that NO also induces Nrf2 nuclear translocation [35]. So far, knowledge of the regulatory mechanisms of Nrf2 activation is very limited. Several studies implied that PI3K is a key regulator of Nrf2 [36,37]. It was also found that protein kinase C (PKC) phosphorylates Nrf2 and regulates concomitant ARE-mediated transcription in response to oxidative stress [38,39]. Mitogen-activated protein kinases (MAPKs), such as ERK1/2 and p38, are also reported to modulate Nrf2 activation [40,41]. Although the signaling pathways of the translocation of Nrf2 were reported in several previous papers [42,43], studies on the signaling pathway of Nrf2 translocation under shear stress stimulation are very limited. Hosoya et al. reported that both laminar and oscillatory shear stresses can trigger the translocation of Nrf2, but only laminar shear stresses can induce Nrf2 binding to the ARE [44]. In this study, we investigated the signaling pathways involved in shear stress-induced Nrf2 translocation.

In a previous study, we showed that shear stress increases intracellular ROS and antioxidant activity in ECs [45]. Generation of ROS may participate in cellular responses and signal transduction. Shear-induced ROS are responsible for inducing HO-1, that has remarkable antioxidant abilities and carries out diverse protective functions in diseases such as atherosclerosis [46-48]. In the present study, we examined the regulatory mechanisms of shear stress on the Nrf2 transcription factor and its downstream target, HO-1, in cultured human umbilical vein endothelial cells (HUVECs). We demonstrated that shear stress induced Nrf2 nuclear translocation, and this process involved PI3K, ROS, and PKC. We also showed that shear stress increased the ARE-binding activity of Nrf2. Parallel experiments using H_2_O_2 _to stimulate ECs were carried out, and similar results were obtained. Our data suggest that in some way, the atheroprotective role of laminar shear stress may be attributed to Nrf2 activation which results in initiation of ARE-mediated gene transcription, including HO-1 expression, that is beneficial to the cardiovascular system.

## Materials and methods

### Cell culture

Primary cultures of HUVECs were harvested from umbilical cord veins by collagenase isolation. The harvested cells were resuspended in culture medium (Medium 199 supplemented with 20% fetal bovine serum (FBS)), plated on 10-cm culture dishes, and then incubated at 37°C in 5% CO_2 _balanced with air. The following day, HUVECs were rinsed with buffer and grown to confluence within 2 to 3 days. The cells were subcultured on fibronectin-coated glass slides. One day prior to the shear stress experiments, the concentration of FBS in the culture medium was reduced to 2%, and this was used throughout the shear stress experiments.

### Shear stress experiments

Exposure of HUVECs to shear stress was conducted in a parallel-plate flow chamber as previously described [49]. Continuous flow of culture medium through the flow chamber was generated by a roller pump. The flow loop system was maintained at 37°C. The pH of the medium was maintained at a constant level by continuous gassing of the medium reservoir with humidified 5% CO_2 _balanced with air.

### Nuclear protein extraction

To prepare nuclear protein extracts, HUVECs were washed with cold phosphate-buffered saline (PBS) and then removed by scraping in detachment buffer (150 mM NaCl, 1 mM EDTA, and 40 mM Tris; pH 7.6). After centrifugation of the cell suspension at 2000 rpm, the cell pellets were resuspended in cold buffer A containing KCl (10 mM), EDTA (0.1 mM), dithiothreitol (1 mM), and phenylmethylsulfonyl fluoride (1 mM) for 15 min. The cells were lysed by adding 10% Nonidet P-40 and then centrifuged at 6000 rpm to obtain a pellet of nuclei. The pelleted nuclei were resuspended in cold buffer B containing HEPES (20 mM), EDTA (1 mM), dithiothreitol (1 mM), and phenylmethylsulfonyl fluoride (1 mM), as well as NaCl (0.4 mM), and then vigorously agitated from time to time, followed by centrifugation. The supernatant containing the nuclear proteins was used for Western blot analysis or an electrophoretic mobility shift assay (EMSA).

### Western blot analysis

Proteins were extracted in sodium dodecylsulfate (SDS) buffer and analyzed by SDS-polyacrylamide gel electrophoresis (SDS-PAGE). After being transferred onto a nitrocellulose membrane, antigens were analyzed by specific antibodies. Antigen-antibody complexes were detected using an ECL detection system (Pierce).

### Reverse-transcriptase polymerase chain reaction (RT-PCR)

The primer of Nrf2 (GenBank accession no.: BC011558) was designed using the GCG system, and the GADPH primer was provided by Dr. H. H. Chen (Institute of Biomedical Sciences, Academia Sinica, Taiwan). Nrf2 primers were synthesized by MB Mission Biotech (Taipei, Taiwan) and consisted of 5'-ACA CGG TCC ACA GCT CAT CAT-3' (forward) and 5'-TTG GCT TCT GGA CTT GGA AC-3' (reverse); GAPDH primers were synthesized by the same company and consisted of 5'-TGG TAT CGT GGA AGG ACT CAT GAC-3' (forward) and 5'-ATG CCA GTG AGC TTC CCG TTC AGC-3' (reverse). When total RNA was isolated from HUVECs, a Superscript III one-step RT-PCR kit was used for the reverse transcription. After 25 cycles, the end products were subjected to 1% agarose electrophoresis to analyze the target RT-PCR products.

### Electrophoretic mobility shift assay (EMSA)

The supernatant containing the nuclear proteins was used for the EMSA. The probe for EMSA was a synthetic 24-mer oligonucleotide (5'-GGG ACT GGT GAC TCA GCA AAA TCT-3') containing the ARE binding site within the promoter region and about 4 kb upstream of the human HO-1 gene [6]. The probe was end-labeled with biotin using the Biotin 3'-end DNA labeling kit from Pierce (Rockford, IL). Both the sense and antisense oligonucleotides were separately labeled and then annealed to form the double-stranded probe. 10 μg of the nuclear protein from either untreated or the different stress-exposed cells was incubated at room temperature for 20 min in binding reaction buffer containing the biotin-labeled 24-mer probe with, or without, an unlabeled competitor. The competitor used was the unlabeled 24-mer just described or the biotin control probe (Pierce). In the antibody supershift assay, the anti-Nrf2 antibody (1 μg, Santa Cruz Biotechnology) was incubated with the mixture for 20 min at room temperature followed by the addition of the biotin-labeled DNA probe. Proteins in the reaction mixture were then separated by electrophoresis in a 5% TBE polyacrylamide gel (Bio-Rad), run at 100 V for 45 min. Protein-DNA complexes were transferred to a positively charged nylon membrane (Bio-Rad) at 100 V for 60 min with 0.5% TBE buffer at 4°C. The transferred DNA was cross-linked to the membrane by 60-s exposure to ultraviolet light. Biotin-labeled DNA was detected by using the LightShift chemiluminescent EMSA kit (Pierce) following the manufacturer's instructions.

## Results

### Shear stress increases Nrf2 expression and nuclear translocation

**Figure 1:**
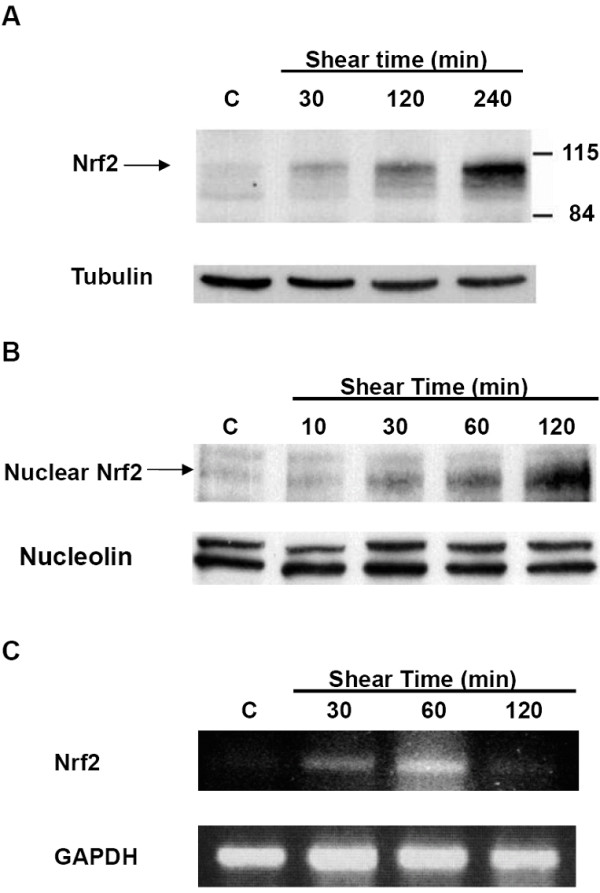
**Shear stress increases Nrf2 protein expression and induces Nrf2 translocation into nuclei**. (A) Shear stress increased the total amount of Nrf2 protein. Human umbilical vein endothelial cells (HUVECs) were exposed to laminar shear stress at a magnitude of 12 dyne/cm^2 ^for the indicated times. Total cell lysates were recovered and subjected to Western blot analysis with anti-Nrf2 and anti-tubulin (internal control, used to indicate equal loading of protein in each lane) antibodies. (B) Shear stress triggered the translocation and accumulation of Nrf2 protein in nuclei. HUVECs were exposed to laminar shear stress (12 dyne/cm^2^) for the indicated times. Nuclear extracts were isolated and subjected to Western blotting with anti-Nrf2 and anti-nucleolin (internal control) antibodies. (C) Shear stress increased Nrf2 mRNA. HUVECs were exposed to laminar shear stress (12 dyne/cm^2^) for the indicated times. Total RNA was isolated, and Nrf2 and GAPDH (internal control) mRNA levels were detected by RT-PCR. Results are representative of three independent experiments.

To examine the effect of shear stress on Nrf2 expression and activation, HUVECs were exposed to a laminar shear stress of 12 dyne/cm^2 ^which resembles physiological blood flow conditions in atherosclerotic plaque-free regions. Shear stress substantially increased the total cellular levels of Nrf2 protein over time (Figure 1A). Despite the fact that the predicted molecular weight of Nrf2 is 66 kD, the major Nrf2 band detected by immunoblotting appeared to be approximately 100 kDa as seen in Figure 1A, similar to results reported in previous studies [50,51].

It had been reported that Nrf2 binds to the cytoskeleton-associated protein, Keap1, in cytoplasm under basal conditions and then is translocated into the nucleus upon stimulation [30]. Herein, we investigated the regulation of Nrf2 activation by shear stress. The nuclear level of Nrf2 protein increased with respect to time in HUVECs subjected to shear stress (Figure 1B), revealing that shear stress induced Nrf2 nuclear translocation, and thus could initiate downstream transcription activities.

Since mRNA of Nrf2 is constitutively expressed in cells [52], we also investigated the mRNA of Nrf2 and found that it exhibited higher expression under shear stress stimulation (Figure 1C). As revealed by RT-PCR, Nrf2 mRNA was elevated after 30 min of shear stress, and reached a maximum at around 60 min. The expression of Nrf2 mRNA was reduced after 120 min of shear stress, but it was still higher than the basal static condition. The results show that shear stress not only induces Nrf2 translocation but also increases the expression of Nrf2 at the mRNA and protein levels.

### Shear stress induces Nrf2 nuclear translocation through a PI3K-dependent pathway

**Figure 2:**
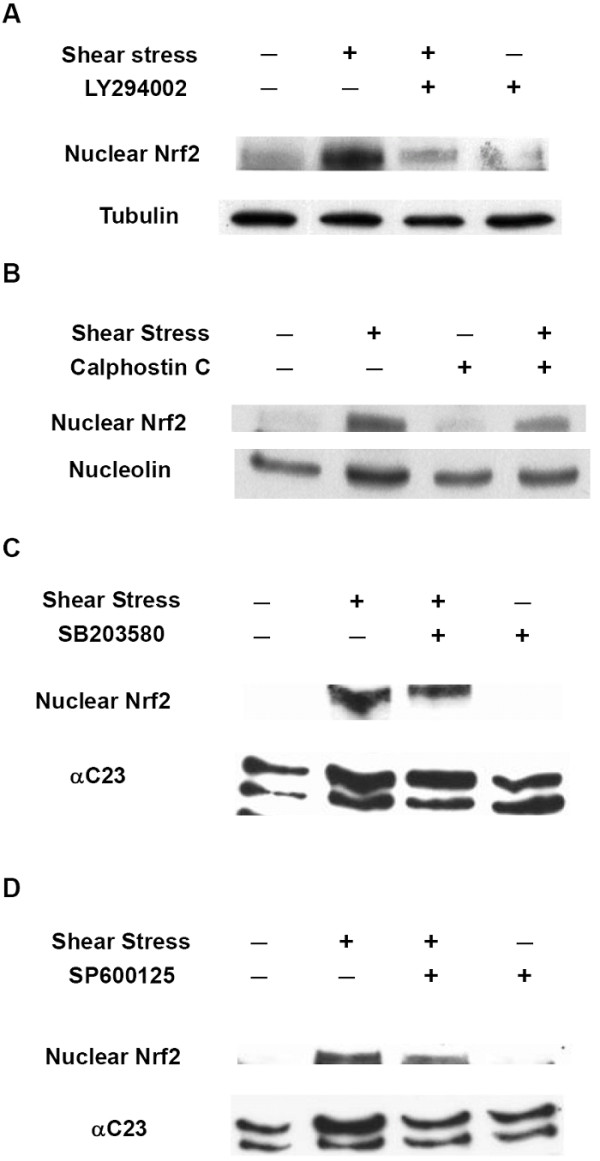
**PI3K is involved in shear-induced Nrf2 nuclear translocation**. (A) PI3K is involved in shear-induced translocation of Nrf2 into nuclei. HUVECs were pretreated with LY294002, a PI3K inhibitor (50 μM), for 60 min and then kept as static controls or exposed to laminar shear stress (12 dyne/cm^2^) in the presence of LY294002 for 120 min. Nuclear extracts were prepared and subjected to Western blotting with an anti-Nrf2 antibody. The cytoplasmic fractions were subjected to Western blotting with an anti-tubulin (internal control) antibody. (B) PKC is involved in Nrf2 activation. HUVECs were pretreated with calphostin C, a PKC inhibitor (200 nM), or DMSO as a negative control for 30 min, and then exposed to laminar shear stress (12 dyne/cm^2^) or kept in a static condition for 2 h. After treatment, nuclear extracts were prepared and subjected to SDS-PAGE and immunoblotted with anti-Nrf2 and anti-nucleolin (internal control) antibodies. Similar results were obtained from repeated experiments. (C) p38 is not involved in shear-induced Nrf2 translocation. HUVECs were pretreated with SB203580, a p38 inhibitor (10 μM), for 30 min, and then kept as a static control or exposed to shear stress in the presence of inhibitors for 60 min. Nuclear extracts were prepared and subjected to Western blotting with anti-Nrf2 and anti-αC23 (internal control) antibodies. Similar results were obtained from repeated experiments. (D) JNK is not involved in shear-induced Nrf2 translocation. HUVECs were pretreated with SP600125, a JNK inhibitor (10 μM), for 60 min, and then kept as a static control or exposed to shear stress in the presence of the inhibitor for 60 min. Nuclear extracts were prepared and subjected to Western blotting with anti-Nrf2 and anti-αC23 (internal control) antibodies. Similar results were obtained from repeated experiments.

Many previous studies implied that Nrf2 activation is mediated through the PI3K pathway [36,37,53]. Since shear stress is known to activate the PI3K pathway, we speculated that PI3K might participate in shear-induced Nrf2 nuclear translocation. As anticipated, we found that shear-induced Nrf2 nuclear translocation was suppressed by pretreating HUVECs with LY294002, a PI3K inhibitor (Figure 2A), suggesting that the PI3K pathway plays a key role in shear-induced Nrf2 activation.

It was found that protein kinase C (PKC) phosphorylates Nrf2 and regulates concomitant ARE-mediated transcription in response to oxidative stress [38,39]. A recent study showed that the anti-inflammatory activity of *Phellinus linteus *(an orange-colored mushroom) is mediated through PKCdelta/Nrf2/ARE signaling that leads to the upregulation of HO-1 [54]. These findings suggest that PKC may play a role in shear-induced Nrf2 nuclear translocation. To test this hypothesis, calphostin C (a PKC inhibitor) was used to pretreat HUVECs prior to the shear stress experiment. Pre-exposure to calphostin C did not affect the basal level of nuclear Nrf2 protein but significantly attenuated shear-induced Nrf2 nuclear translocation (Figure 2B), indicating that PKC is involved in the nuclear translocation of Nrf2 in response to shear stress.

Chen et al. determined that dietary chemopreventive compounds induce Nrf2 translocation through MAPK pathways [55]. Owuor et al. also reported similar pathways for Nrf2 translocation under chemical stress [56]. Many MAPK family proteins, including p38 and JNK, are activated by shear stress stimulation. It is likely that the translocation of Nrf2 under shear stress conditions is regulated by the MAPK pathway. However, the use of SB203580 (a p38-specific inhibitor) and SP600125 (a JNK inhibitor) did not attenuate the translocation of Nrf2 (Figs. 2C &2D). This suggests that even though MAPK pathways are activated by shear stress, p38 and JNK are not involved in shear-induced Nrf2 translocation.

### Shear stress increases Nrf2 nuclear accumulation and binding activity to ARE through ROS

**Figure 3:**
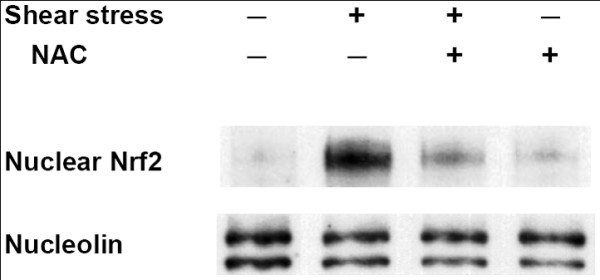
**ROS are necessary for Nrf2 nuclear translocation**. HUVECs were pretreated with NAC, a reactive oxygen species (ROS) scavenger (10 mM), for 30 min and then kept as a static control or exposed to shear stress (12 dyne/cm^2^) in the presence of NAC for 30 min. Nuclear extracts were prepared and subjected to Western blotting with anti-Nrf2 and anti-nucleolin (internal control) antibodies. Similar results were obtained from repeated experiments.

In a previous study we showed that shear stress increases intracellular ROS which participate in cellular responses and signal transduction in endothelial cells [45]. Since Nrf2 is a gene that can be stimulated by oxidants and electrophiles [33,34], we also speculated that ROS might participate in shear-induced Nrf2 nuclear translocation. We found that shear-induced Nrf2 nuclear translocation could be suppressed by pretreating endothelial cells with N-acetyl cysteine (NAC), an ROS scavenger, suggesting that ROS are essential for shear-induced Nrf2 activation (Figure 3). We also found that pretreating cells with NAC had an inhibitory effect on Nrf2 mRNA and protein levels (data not shown).

**Figure 4:**
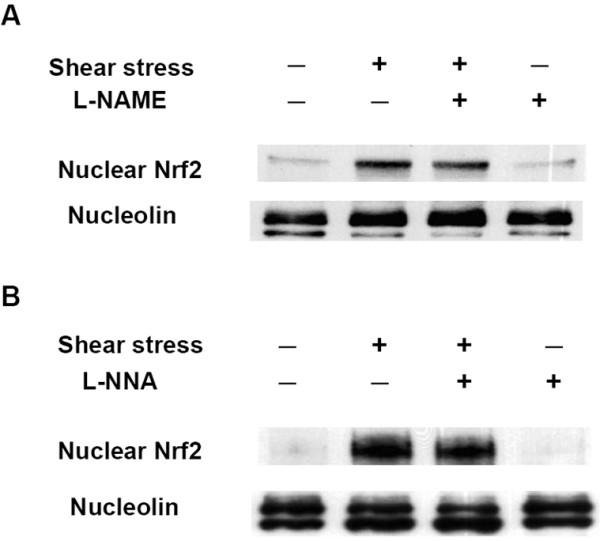
**Shear-induced Nrf2 nuclear translocation is not affected by nitric oxide synthase (NOS) inhibitors**. (A and B) Shear-induced NO is not involved in the Nrf2 nuclear translocation. HUVECs were (A) pretreated with L-NAME, an NOS inhibitor (300 μM), for 1 h or (B) pretreated with L-NNA, an NOS inhibitor (250 μM), for 1 h, and then kept as a static control or exposed to shear stress in the presence of the inhibitor for 2 h. Nuclear extracts were prepared and subjected to Western blotting with anti-Nrf2 and anti-nucleolin (internal control) antibodies. Similar results were obtained from repeated experiments.

On the other hand, a recent study demonstrated that NO can also induce Nrf2 nuclear translocation [35]. It is known that shear stresses increase NO production in endothelial cells [57,58]. We thus investigated if NO plays any role in shear-induced Nrf2 nuclear translocation. Using eNOS inhibitors (L-NAME and L-NNA) to block NO production in endothelial cells, no effect was seen on shear-induced Nrf2 nuclear translocation (Figures 4A &4B). Our results thus indicate that shear-increased NO production is unimportant for the nuclear translocation of Nrf2 induced by shear stress.

**Figure 5:**
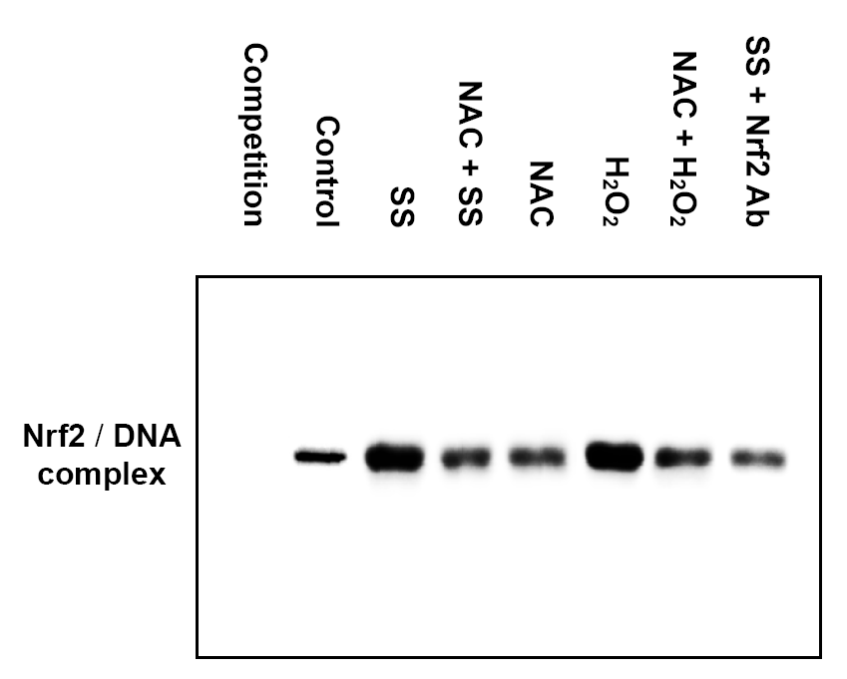
**Shear stress increases the antioxidant response element (ARE)-binding activity of Nrf2 through reactive oxygen species (ROS)**. HUVECs were kept in a static condition (Control), exposed to shear stress (SS) of 12 dyne/cm^2 ^for 2 h, incubated with H_2_O_2 _(200 μM) for 2 h (H_2_O_2_), or pretreated with NAC (10 mM) (NAC) and then incubated with H_2_O_2 _(200 μM) for 2 h (NAC+H_2_O_2_). Before applying the shear stress, HUVECs were pretreated with NAC (10 mM) for 60 min and then exposed to shear stress for 2 h (NAC+SS). Total nuclear extracts were prepared and analyzed by EMSA using a biotin-labeled oligonucleotide probe containing Nrf2 consensus binding sites corresponding to the HO-1 promoter region (5'-GGG ACT GGT GAC TCA GCA AAA TCT-3', within the promoter region lying about 4 kb upstream of the human HO-1 gene). The specificity of the Nrf2 binding was assessed by preincubating nuclear extracts with the biotin-labeled oligonucleotide probe in the presence of 100× unlabeled oligonucleotide probe to compete for Nrf2 binding (Competition). EMSA performed on the nuclear extracts preincubated with an Nrf2 antibody (SS+Nrf2 Ab) was also included. Results are representative of duplicate experiments with similar results.

Nrf2 is a transcription factor that is essential for the ARE-mediated induction of many redox-sensitive genes including HO-1. To investigate the effect of shear stress on the ARE-binding activity of Nrf2, nuclear proteins were isolated from endothelial cells and used for an EMSA. An oligonucleotide (5'-GGG ACT GGT GAC TCA GCA AAA TCT-3') containing the ARE-binding site within the promoter region lying about 4 kb upstream of human HO-1 gene was used as a probe in the EMSA [6,59]. In the antibody supershift assay, the anti-Nrf2 antibody was incubated with nuclear proteins prior to the addition of the oligonucleotide. The results indicated that shear stress enhanced Nrf2 binding activity to ARE, and this effect was significantly repressed by the ROS scavenger, NAC (Figure 5). Similarly, H_2_O_2 _also increased the ARE-binding activity of Nrf2, and this event was significantly suppressed by NAC (Figure 5). Based on these findings, it is suggested that shear stress promotes the binding activity of Nrf2 to ARE through ROS.

### Shear stress and H_2_O_2 _induce HO-1 protein expression through the PI3K pathway

**Figure 6:**
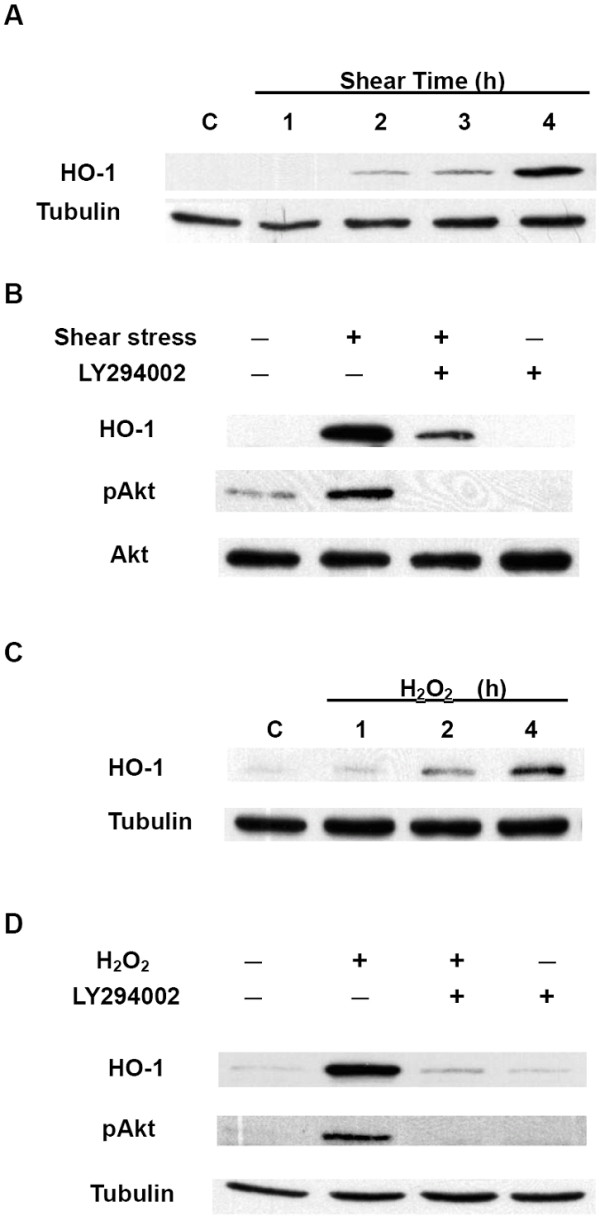
**Shear stress and hydrogen peroxide induce HO-1 expression via a PI3K-dependent pathway**. (A) Shear stress increased HO-1 protein expression. HUVECs were exposed to laminar shear stress (12 dyne/cm^2^) for the indicated times. After cell lysis, total cell lysates were prepared and subjected to Western blotting with anti-HO-1 and anti-tubulin (internal control) antibodies. (B) PI3K is involved in shear-induced HO-1 protein expression. HUVECs were pretreated with LY294002, a PI3K inhibitor (50 μM) for 60 min and then kept as static controls or exposed to shear stress in the presence of LY294002 for 4 h. Total cell lysates were subjected to Western blotting with anti-HO-1, anti-phospho-Akt, and anti-Akt (internal control) antibodies. (C) Hydrogen peroxide (H_2_O_2_) increased HO-1 protein expression. HUVECs were incubated with H_2_O_2 _(200 μM) for the indicated times. Total cell lysates were subjected to Western blotting with anti-HO-1 and anti-tubulin (internal control) antibodies. (D) PI3K is involved in H_2_O_2_-induced HO-1 protein expression. HUVECs were either kept as a static control or treated with H_2_O_2 _(200 μM) for 4 h or pretreated with LY294002, a PI3K inhibitor (50 μM), for 60 min. Total cell lysates were subjected to Western blotting with anti-HO-1, anti-phospho-Akt, and anti-tubulin (internal control) antibodies. Results are representative of duplicate experiments with similar results.

As presented earlier, PI3K plays a vital role in shear-induced Nrf2 activation (Figure 2). Activated Nrf2 was demonstrated to bind to an ARE-containing oligonucleotide probe (Figure 5), and this might trigger the expression of ARE-containing genes including HO-1. As expected, shear stress increased HO-1 protein levels in HUVECs in a time-dependent manner (Figure 6A), and this event was significantly suppressed by pretreating HUVECs with LY294002 (a PI3K inhibitor), thus indicating an essential role of PI3K in shear-induced HO-1 protein expression (Figure 6B). H_2_O_2 _also increased HO-1 protein levels in a time-dependent manner (Figure 6C), and it was significantly repressed by pretreating HUVECs with LY294002 (a PI3K inhibitor), indicating that PI3K also plays a key role in H_2_O_2_-induced HO-1 protein expression (Figure 6D). Taken together, our data suggest that shear stress and hydrogen peroxide both induce HO-1 protein expression through the PI3K pathway.

## Discussion

In the present study, we examined the regulatory mechanisms of shear stress on the Nrf2 transcription factor and its downstream target, HO-1, in HUVECs. It was found that shear stress activated Nrf2 nuclear translocation and increased the amount of Nrf2 protein and the level of mRNA transcription (Figure 1). The translocation of Nrf2 occurred after 10 min of shear stress, suggesting that Nrf2 translocation which induces downstream gene regulation is a rapid response. However, the mRNA level of Nrf2 increased obviously after 30 min of shear stress and reached a maximum at around 60 min. Hong et al. and Eggler et al. reported that under a basal condition, Nrf2 undergoes Cul3-dependent ubiquitination and proteosomal degradation, but under various stimuli, Keap1 is ubiquitinated to release Nrf2 into the nucleus [60,61]. It is likely that when HUVECs are exposed to shear stress, the early response is ubiquitination of Keap1 which induces Nrf2 translocation. Nuclear translocation may decrease the concentration of Nrf2 in the cytosol thus triggering the expression of Nrf2 at both the mRNA and protein levels. But by 2 h later, the cells may have adapted to the shear stress stimulation, and thus the transcription level of Nrf2 began to decline. However, the amount of Nrf2 protein was still higher than the basal condition (Figure 1A).

We also found that PI3K played a crucial role in shear-induced Nrf2 translocation (Figure 2). Kang et al. revealed that before entering nuclei, it is necessary for Nrf2 to be translocated to plasma membranes where it is phosphorylated by PI3K or PKC [36,42]. Based on our results, we found that Nrf2 translocation and activation were PI3K-dependent. But PI3K is a lipid kinase, and the substrate of PI3K should be a lipid like PIP2, not Nrf2. This implies that the mechanism of Nrf2 translocation is indirectly mediated by PI3K. There are likely more protein kinase(s) involved in Nrf2 activation, and PKC may be one candidate. When shear stresses activate PI3K, it phosphorylates PIP2 to PIP3, and at this time, phosphoinositide-dependent kinase 1 (PDK1) may bind to PIP3 through its PH domain. PKC, one of the downstream substrates of PDK1, can be phosphorylated and activated. Activation of Nrf2 by PKC possibly occurs through the phosphorylation of S40 at the Neh2 domain of Nrf2, which interacts with Keap1 [38,39]. The phosphorylation of S40 triggers a conformational change in Nrf2, and thus Nrf2 is released from Keap1. Free Nrf2 in the cytosol is translocated into nuclei or interacts with other protein kinase(s). We speculate that nuclear translocation of Nrf2 requires either phosphorylation by PKC or another serine or threonine kinase at S40 of the Neh2 domain, which disrupts the interactions of Nrf2 and Keap1, leading to Nrf2 separating from Keap1. With the exception of PI3K, ROS also participate in the nuclear translocation of Nrf2 (Figure 3). However, the detailed mechanism is not clear at the present time. ROS may change the intracellular redox state of HUVECs, triggering the formation of disulfide bonds between the SH groups of cysteine residues within protein molecules, thus altering the activities of those proteins (see discussion below).

In a previous study, we showed that shear stresses increase the amount of ROS in endothelial cells [45]. The generation of ROS may participate in many cellular responses and signal transduction. This study verified that ROS are important regulators of the nuclear translocation of Nrf2. The intracellular redox state of HUVECs can be altered by ROS. On the other hand, many previous studies reported that shear stresses activate eNOS and increases the amount of endogenous NO [57,58], which also affects the intracellular redox state of HUVECs. However, we found that shear-increased NO had no influence on Nrf2 translocation (Figure 4).

The detailed mechanism of the regulation of Nrf2 translocation by the intracellular redox state remains to be elucidated. Wakabayashi et al. showed that both C273 and C288 residues are necessary for Keap1 to repress Nrf2 [62]. Based on our data in Figs. 3 &4, we speculated that the redox state of HUVECs may influence Nrf2 translocation by changing the redox state of the C273 and C288 residues of Keap1. There might be several possible mechanisms that alter the C273 and C288 residues of Keap1 and thus release Nrf2. For example, it is possible that when the amount of ROS is increased, the SH groups on C273 and C288 may be oxidized to form disulfide bonds, thus triggering Keap1 to release Nrf2 and initiating Nrf2 nuclear translocation. It was reported that an elevated concentration of xenobiotics in the cytosol causes oxidative stress and induces the dissociation of Nrf2 from Keap1 through the above-mentioned ROS-mediated mechanism [31]. Another possibility is the nitrosylation of C273 and C288 residues of Keap1 which causes the release of Nrf2, and this mechanism is carried out only when the concentration of NO in the cytosol is much higher than the basal condition. We speculated that the concentration of shear-induced NO was not high enough to trigger Nrf2 translocation. Thus, it is likely that the ROS-mediated mechanism plays a relatively more important role than the NO-mediated mechanism in regulating Nrf2 translocation.

As for the intracellular sources of ROS, Li et al. and Hancock et al. showed that NADPH oxidases (NOXs) are a major source of ROS [63,64], and in our preliminary study, we found that the use of small interfering (si)RNA against NOX2 repressed Nrf2 translocation (data not shown). This result also supports our deduction that a change in the redox state of HUVECs triggers Nrf2 translocation.

Results obtained from the gel shift assay further revealed that shear stress enhanced the ARE-binding activity of Nrf2 through the involvement of ROS (Figure 5). Shear-induced HO-1 protein expression was also suppressed by a PI3K inhibitor (Figure 6). Parallel experiments were conducted using H_2_O_2_, a major ROS, as the stimulus, and similar results were obtained. These results provide evidence that shear-induced Nrf2 regulates HO-1 expression via binding to the ARE in the promoter region, and this regulation involves PI3K and ROS.

## Conclusion

In the present study, we examined the regulation of shear stress on Nrf2 in HUVECs. We demonstrated that a laminar shear stress of 12 dyne/cm^2 ^induced Nrf2 nuclear translocation, and we found that PI3K, PKC, and ROS, but not MAPKs (p38 and JNK), were involved in the signaling pathway. We also found that shear stress increased the ARE-binding activity of Nrf2 and HO-1 expression. HO-1 has been shown to have remarkable antioxidant abilities and is responsible for diverse protective functions against diseases such as atherosclerosis. Our data suggest that the atheroprotective role of laminar shear stress is partly attributed to Nrf2 activation which results in ARE-mediated gene transcription, including HO-1 gene expression, which is beneficial to the cardiovascular system.

## Abbreviations

**ARE**: antioxidant response element; **ECs**: endothelial cells; **eNOS**: endothelial nitric oxide synthase; **HO-1**: heme-oxygenase 1; **HUVECs**: human umbilical vein endothelial cells; **MAPKs**: mitogen-activated protein kinases; **NAC**: N-acetyl cysteine; **NO**: nitric oxide; **Nrf2**: nuclear factor erythroid 2-related factor 2; **PI3K**: phosphatidylinositol 3-kinase; **PKC**: protein kinase C; **ROS**: reactive oxygen species.

## Competing interests

The authors declare that they have no competing interests.

## Authors' contributions

CYH and HYH contributed equally to this study. CYH and HYH designed and performed the experiments, participated in the discussion of the results, and drafted part of the manuscript. WYW performed the inhibitor experiments and participated in the discussion of the results. CAL performed the EMSA experiments, participated in the discussion of the results, and helped to draft part of the manuscript. YCT participated in the experimental design and discussion of the results, and helped to draft part of the manuscript. YJC coordinated the experiments and participated in the discussion of the results. DLW co-conceived and co-designed the study, participated in the experimental design and interpretation of the results, reviewed and helped to revise the manuscript. HJH conceived and designed the study and the experiments, coordinated the execution of the study, interpreted the results, wrote the manuscript and also revised it. All authors read and approved the final manuscript.
